# Advances in cotton harvesting aids

**DOI:** 10.3389/fpls.2025.1570251

**Published:** 2025-04-24

**Authors:** Zhangshu Xie, Xuefang Xie, Yeling Qin, Dan Yang, Zhonghua Zhou, Qiming Wang, Aiyu Liu, Xiaoju Tu

**Affiliations:** ^1^ Cotton Research Institute, Agronomy College, Hunan Agricultural University, Changsha, China; ^2^ Rubber Research Institute, Chinese Academy of Tropical Agricultural Sciences, Haikou, China; ^3^ Planting (Farming) Development Management Unit, Jingzhou County Agriculture and Rural Bureau, Huaihua, China; ^4^ College of Biological Science and Technology, Hunan Agricultural University, Changsha, China

**Keywords:** cotton, defoliation, ripening, harvesting aids, research progress and directions

## Abstract

During the cotton harvesting stage, the application of chemical harvest aids, such as thidiazuron and ethephon, facilitates cotton defoliation and boll maturation, serving as a crucial management tool in modern cotton cultivation systems. This paper reviews recent advancements in cotton defoliation and ripening research; delves into the physiological mechanisms underlying defoliation, boll maturation, and cotton fiber development; and summarizes the effects of major defoliants and herbicide-type desiccants on plants. It also explores the roles of hormones and genes that are involved in the defoliation process and identifies the key factors influencing the effectiveness of harvest aids. Additionally, this paper offers recommendations and scientific prospects for optimizing cotton defoliation and ripening technologies in the future. Through these contributions, it aims to provide valuable insights for the research and application of efficient harvesting of mature cotton, stimulate innovation in cotton defoliation and ripening technologies, enhance the quality and yield of cotton, reduce labor costs, and contribute to the sustainable development of the cotton industry.

## 1 Introduction

Cotton (*Gossypium hirsutum* L.) is an important economic crop and widely cultivated globally (Yu et al., 2022). As a perennial woody plant, cotton exhibits unlimited growth potential, characterized by prominent concurrent vegetative and reproductive growth (Liu, 2020). Its leaves, which are vital nutritional organs, undergo defoliation, an essential physiological process in the cotton lifecycle. This process encompasses various types of leaf fall: the first is natural defoliation, driven by the natural reproductive maturation and senescence of cotton, representing a normal physiological response of the plant; the second is unnatural defoliation, often caused by biotic (such as pest and disease infestations) or abiotic (e.g., extreme environmental conditions) stresses. In cotton production management, a form of regulated defoliation can also be achieved through the application of defoliants or other chemical agents (Mishra et al., 2023). Among these, the use of defoliant accelerators as a cotton cultivation management measure can optimize the growth pattern of cotton by effectively curbing excessive vegetative growth, thus promoting the allocation of nutrients to reproductive organs (bolls) and thereby accelerating boll maturation and opening (Liu et al., 2020). Furthermore, the full mechanization of cotton production is an inevitable development in the industry, with defoliation being a critical step in mechanized harvesting (Zhu et al., 2018). The application of defoliant accelerators can resolve the issue of inconsistent maturation caused by late maturation due to excessive vegetative growth; balance the reduction of early boll senescence and degradation during the initial development stage with the promotion of boll maturation in the later stages; fully utilize favorable meteorological conditions to enhance the cotton fiber quality; and effectively reduce the potential hazards of adverse autumn environments on bolls that are about to open, thereby minimizing natural weathering losses caused by seasonal changes. Additionally, it maximizes and increases the leaf fall rate during the boll opening period, reduces leaf contamination of cotton fibers, and lowers the impurity rates in cotton fibers during mechanized harvesting, which is of significant importance for improving the yield and quality of cotton (Zhang et al., 2019). With continuous advancements in technology, cotton defoliation techniques have been fully applied and refined. However, due to the susceptibility of defoliant accelerators to natural climatic conditions (temperature, rainfall, sunlight, etc.), agricultural management practices, cultivars, and defoliant application methods, the efficacy of defoliation varies among cotton plants, leading to numerous issues and challenges in cotton defoliation (Fan, 2008; Zhou et al., 2020). This paper systematically summarizes the recent progress in applied research from the perspectives of the mechanisms of defoliant accelerators and the factors affecting their efficacy, incorporating the roles of related hormones and genes. Our aim is to provide theoretical foundations and methodological references for related studies.

## 2 Effect of chemical harvesting aids on cotton

In most cases, cotton leaf abscission and boll maturation and splitting are closely associated with the regulatory effects of ethylene (i.e., the antagonistic interactions between ethylene and auxin). Consequently, chemicals that induce ethylene production often simultaneously possess defoliating and maturation-promoting functionalities (Chinese Academy of Agricultural Sciences, Cotton Research Institute, 2013). In international agricultural practice, these are collectively known as chemical harvest aids. These aids also encompass inhibitors that suppress the regeneration of axillary and apical buds, as well as desiccants that remove plant moisture. The function of desiccants is to effectively promote and accelerate the wilting and drying of leaves and weeds that do not naturally abscise, even after the application of defoliants prior to harvest, thereby enhancing the harvest efficiency and crop quality (Chinese Academy of Agricultural Sciences, Cotton Research Institute, 2013). As a category of plant growth regulators (PGRs), various chemical harvest aids are currently extensively applied in cotton cultivation management practices, including defoliation, the suppression of regeneration, the promotion of boll maturation and fiber shedding, and weed control. **Table 1** presents an overview of the effects of different harvest aids on cotton’s growth and development.

**Table 1:** Evaluation of the effects of different harvesting aids on the growth and development of cotton.

Chemical name	Effect				
	Removal of mature foliage	Removal of juvenile foliage	Promotion of boll ripening and cracking	Regrowth suppression	Acts as a desiccant for weeds
Ethephon	Fair–Good	Fair	Excellent	Poor	Poor
Ethephon + Urea sulfate	Good	Good	Excellent +	Poor	Fair
Ethephon + Cyclanilide	Good–Excellent	Fair–Good	Excellent +	Fair	Poor
Paraquat	Fair	Fair	Poor–Fair	Poor	Good
Protoporphyrinogen oxidase inhibitors	Good	Fair	Poor	Poor	Fair
Sodium chlorate	Fair	Poor	Poor	Poor	Fair–Good
Thidiazuron	Good–Excellent	Good	Poor	Good–Excellent	Poor
Thidiazuron + Diuron	Good–Excellent	Good	Poor	Good–Excellent	Poor
Tribufos	Good–Excellent	Poor–Fair	Poor	Poor	Poor

This table is modified from reference (Kemerait, 2021).

Edmisten suggests that defoliants, maturation agents, and desiccants should generally be applied at least 14 days before the target harvest period to effectively increase the rate of leaf abscission and drying, thereby promoting boll maturation and splitting (Edmisten, 2012). Applying defoliation and maturation agents before harvesting cotton can promote the reproductive growth of cotton plants, stimulate early boll maturation and splitting, facilitate the concentration of fiber shedding in bolls, and reduce the risk of the cotton being affected by adverse weather conditions (Siebert and Stewart, 2006). This enhances the mechanical harvesting efficiency, reduces the content of leaves and other impurities in harvested cotton fibers, and prevents the moisture that is carried by cotton leaves from affecting the dryness of the fiber, thereby improving its quality. It also extends the harvesting period, reduces boll rot, and decreases the degree of plant lodging. In cases where pests and diseases are prevalent during the harvest period, spraying defoliation and maturation agents can block the spread of diseases and prevent pests from feeding on the reproductive organs of cotton plants, thereby reducing yield losses (Chu et al., 1992). In cotton fields with dense weed growth, desiccant-active herbicides can also improve ventilation and light penetration in the lower canopy of cotton, thereby promoting fiber shedding and increasing the harvest efficiency (Faircloth et al., 2009). Agents that act on leaves to promote their abscission are generally referred to as defoliants, while those that promote boll maturation and fiber shedding are called maturation agents. Meanwhile, desiccants primarily target grasses (weeds) or other plant leaves. For ease of expression, this paper will collectively refer to them as harvest aids.

### 2.1 Physiological process of cotton leaf abscission

The abscission of plant organs, including leaves, petals, whole flowers, young fruits, and even stems, is a strictly regulated biological phenomenon. Organs undergo abscission in response to their inherent physiological functions or to biotic and abiotic stresses and damage. The core of the phenomenon of abscission lies in the formation of specific regions called abscission zones (AZs). Depending on the plant species and the organ involved, an AZ typically consists of 5-50 layers of specialized cells (Patterson, 2001). As a key sensing site, the AZ can integrate various developmental signals and external stimuli, triggering changes in gene expression patterns and thereby regulating the abscission process. The abscission process can be divided into three stages: the first stage is signal perception and transduction, involving the recognition and transmission of signals that are necessary for cell differentiation and AZ formation; the second stage is regulation, which includes hormone-mediated regulation of the abscission process and is accompanied by the specific expression of a series of abscission-responsive genes; the final stage is execution and separation, during which the connections between AZ cells gradually loosen, ultimately leading to the separation and abscission of the organ or tissue (Pautot et al., 2025). The dynamic balance between ethylene and auxin in the AZ plays a central regulatory role in the abscission process, with ethylene acting as a positive regulator that accelerates abscission, while auxin delays or inhibits abscission. There is diversity in the patterns and types of organs that undergo abscission among different species, indicating the adaptive responses of plants to varying environmental conditions during evolution (Li and Su, 2024).

An AZ is established in the region where the cotton petiole connects to the stem, resulting in the detachment of leaves from the plant. Harvest aids generally facilitate leaf abscission through two mechanisms: either indirectly promoting AZ formation by damaging the leaf’s green tissues or directly inducing AZ formation, thereby causing leaf detachment (Geetha, 2021). Leaf abscission is typically accompanied by alterations in the leaf water potential and a reduction in chlorophyll content (Primka and Smith, 2019). The cotton leaf abscission process is associated with increased ethylene biosynthesis in the AZ and heightened cellulase activity. Chemical agents such as ethephone, carfentrazone, thidiazuron (TDZ), and chlorate are commonly employed in cotton cultivation for defoliation. These chemicals elevate the ethylene levels within the leaves and induce the formation of abscisic acid (ABA) at the junction between the petiole and the branch, thereby accelerating leaf senescence and abscission (Taylor et al., 1993).

**Figure 1:**
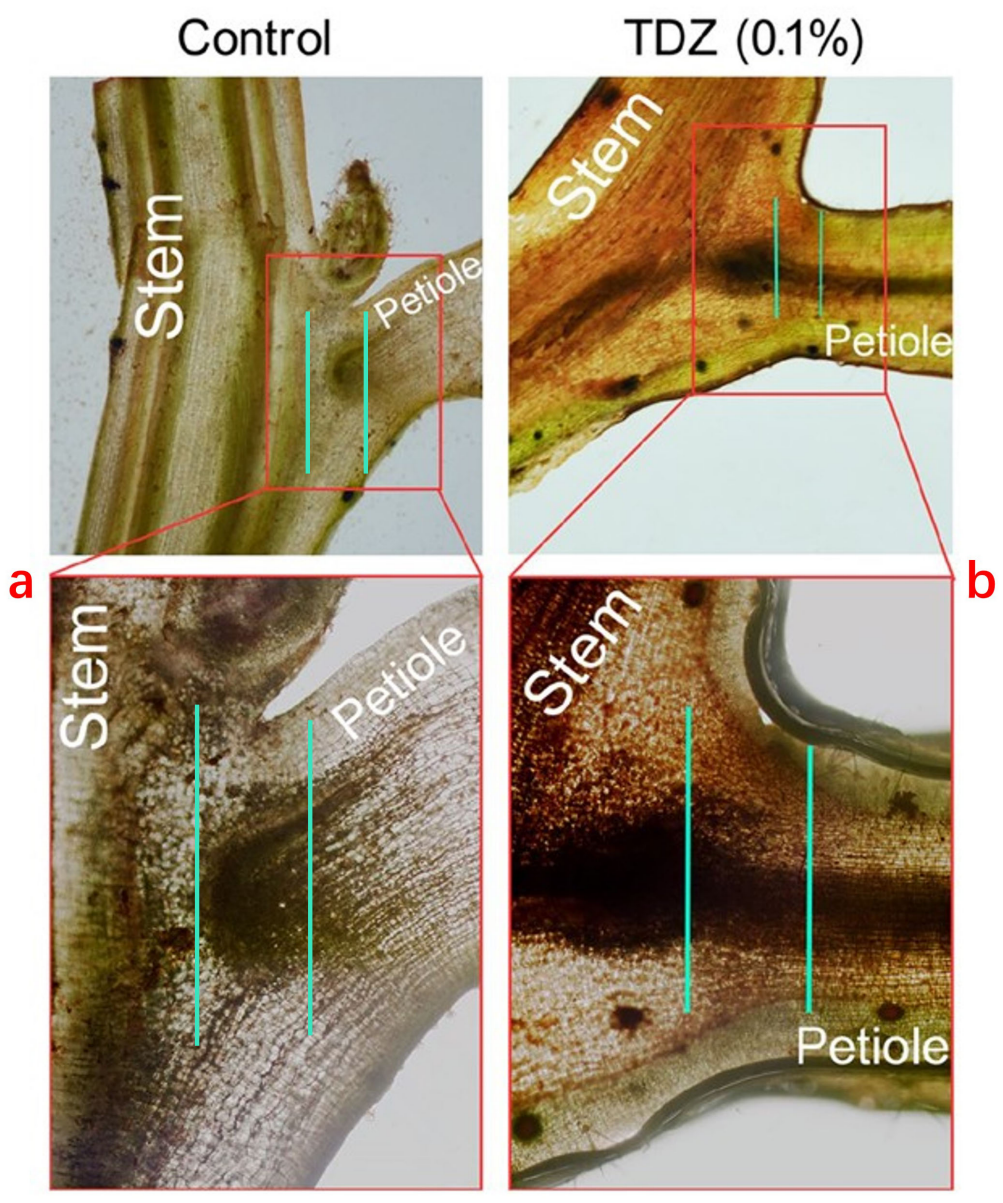
Changes after staining of leaves treated with clear water (control) and TDZ (0.1%) for 4 days using DAB (Diaminobenzidine). **(a)** No abscission zones and no enrichment of H_2_O_2_ were found. **(b)** The abscission zones are present, and H_2_O_2_ is enriched. The figure is modified from reference (Li et al., 2021).

Ethephon increases the production of endogenous ethylene synthase in cotton leaves and enhances the synthesis of the AZ-specific cellulase *GhCel1* in the leaves. Pre-treating cotton leaf explants with the ethylene inhibitor 1-MCP (1-methylcyclopropene) can significantly delay the occurrence of this abscission phenomenon (Mishra et al., 2008). The effect of harvest aids on cotton leaf abscission also involves the accumulation of H_2_O_2_. Taking TDZ as an example, it induces non-biological stress in cotton leaves, leading to cell dehydration, oxidative stress damage, or cell death, which in turn triggers extensive leaf abscission. The accumulation of H_2_O_2_ was detected in the AZs of TDZ-treated cotton leaves, and high concentrations of TDZ (0.1%) cause sustained H_2_O_2_ production (**Figure 1**). Research indicates that RBOH (respiratory burst oxidase homolog) is one of the main pathways through which plants generate H_2_O_2_ in response to environmental stress (Marino et al., 2012). Using the RBOH inhibitor DPI (diphenyleneiodonium chloride) effectively inhibits the leaf abscission caused by TDZ in cotton, suggesting that RBOH-derived H_2_O_2_ is part of the abscission signaling in the AZ.

*GhCel1* is a crucial cellulase gene in cotton leaf abscission. It facilitates the hydrolysis of cellulose in the cell wall, leading to the disruption of cell adhesion and promoting leaf detachment (Mishra et al., 2008). Research indicates that *GhCel1* expression is significantly upregulated under the influence of defoliants such as ethylene, TDZ, and Coronatine (COR), especially in the AZ, where the activity of hydrolytic enzymes, including cellulase and polygalacturonase, is enhanced, thus driving the breakdown of the cell wall. The *GhCel1* expression is also regulated by plant hormones, including ethylene, cytokinin, and abscisic acid, with ethylene playing a dominant role. Additionally, the expression of *GhCel1* varies depending on the defoliant used, with the highest expression being observed under COR treatment. By facilitating cell wall hydrolysis, *GhCel1* accelerates leaf abscission, which plays a critical role in improving the efficiency of mechanical cotton harvesting and enhancing the quality of cotton (Su and Finlayson, 2012).

Additionally, both biotic and abiotic stresses can trigger the accumulation of reactive oxygen species (ROS), which is a key mechanism leading to oxidative damage and cell death in plants (Peláez-Vico et al., 2024). Excessive ROS production in the AZ activates cell-wall-degrading enzymes, causing cell wall dissolution. At the same time, ROS induce programmed cell death (PCD) in the AZ, leading to subsequent leaf abscission (Bar-Dror et al., 2011). Endogenous ROS in plants can also be regulated by antioxidant enzymes with ROS-scavenging abilities, such as superoxide dismutase (SOD) and catalase (CAT) (Averill-Bates, 2024). In cassava, the co-overexpression of SOD and CAT1 reduces the levels of endogenous H_2_O_2_ in the AZ under water-deficient conditions, delaying the separation of AZ cells. This indicates that the plant antioxidant system plays an important role in regulating leaf abscission by modulating endogenous ROS levels (Liao et al., 2016).

Photosynthesis occurring in the leaves converts light energy into chemical energy and stores it in carbohydrate molecules (sugars). Studies have shown that leaf abscission in cotton under TDZ treatment is related to changes in photosynthesis. After 24 hours of TDZ treatment, excessive ROS production and cellular structural damage prevent photosynthesis from occurring in the leaves (Xu and Rothstein, 2018). Moreover, TDZ rapidly and significantly reduces the net photosynthesis (Pn), transpiration rate (Tr), and stomatal conductance (Gs) of cotton leaves, and there is a positive correlation between these photosynthetic parameters and the rate of leaf abscission. Under TDZ treatment, the expressions of genes associated with photosynthesis, chlorophyll metabolism, and carbon storage fixation mechanisms in the leaves are generally downregulated, which is consistent with the gene expression patterns that can be observed during natural leaf senescence (Jin et al., 2020).

Furthermore, recent research indicates that TDZ specifically enhances the accumulation of brassinosteroids and jasmonic acid in cotton leaves. A total of 13,764 genes are differentially expressed under TDZ treatment, with the synthesis, metabolism, and signal transduction pathways of auxin, cytokinin, and brassinosteroids all participating in the TDZ-induced leaf abscission process. Among these, eight auxin transport genes (*GhPIN1-c_D*, *GhPIN3_D*, *GhPIN8_A*, *GhABCB19-b_A*, *GhABCB19-b_D*, *GhABCB2-b_D*, *GhLAX6_A*, and *GhLAX7_D*) exhibit specific responses to TDZ treatment. Additionally, a weighted correlation network analysis (WGCNA) identified five core transcription factors (*GhNAC72*, *GhWRKY51*, *GhWRKY70*, *GhWRKY50*, and *GhHSF24*) that play pivotal roles in the TDZ-mediated chemical defoliation process (Liao et al., 2023).

In cotton production practices, applying a single defoliant may achieve satisfactory leaf abscission from an economic perspective. However, under less-than-ideal defoliation conditions, mixing it with other chemical substances often yields more desirable results to control the growth of regrowth leaves. For example, a mixture of ethephon and TDZ can significantly enhance cotton leaf abscission and boll maturation (Du et al., 2013). For late-sown cotton, the defoliation efficiency of ethephon combined with TDZ is higher than that of ethephon combined with chlorpyrifos or thiophanate (Gwathmey and Hayes, 1997).

Incorporating additional adjuvants into defoliant formulations not only enhances the efficacy of defoliation and maturation, enabling cotton bolls to mature and disperse fibers earlier, but also mitigates the incidence of pests, diseases, and plant lodging (Cathey, 1986). Research dating back to the 1950s demonstrated that the inclusion of certain adjuvants could significantly increase the effectiveness of defoliants: the addition of nonionic surfactants (NISs) to sodium chlorate and monosodium cyanamide increased both the volume and rate of leaf abscission, including the detachment of withered yet unfallen leaves (Brown, 1957). Other adjuvants that are used include crop-oil concentrate (COC), Prep (a nonionic surfactant), and ammonium sulfate (Jones et al., 1999).

### 2.2 Physiological processes of cotton boll maturation and fiber development

The application of defoliants is intended to enhance the defoliation efficiency, primarily to accelerate the maturation of cotton bolls (Faircloth et al., 2009). These chemical agents are commonly known as “boll openers.” As cotton bolls mature and approach the cracking stage, the vascular tissues at the base of the boll stalk differentiate into a corky layer, effectively preventing moisture from infiltrating the boll’s interior. Additionally, a noticeable dissociation occurs between the inner layer of these vascular tissues and the carpels (boll shells), followed by a dehydration process that further weakens and separates the boll structure, ultimately resulting in the natural cracking of the boll (Chinese Academy of Agricultural Sciences, Cotton Research Institute, 2013). Boll cracking necessitates the complete dehydration of the boll, a process that is regulated by ethylene. Ethephon, an ethylene precursor, is utilized as both a defoliant and a boll opener in cotton cultivation. Similarly to leaf abscission in cotton, the maturation and cracking of bolls are fundamentally driven by the ripening action of ethylene. Throughout the progression from the initial onset of cracking to the full emergence of lint, the release of ethylene follows a specific pattern at various developmental stages. When the boll shell undergoes slight cracking (the linear cracking stage), the ethylene release begins to increase markedly; as the cracking intensifies to the stage of noticeable fissures (the microcracking stage), the ethylene release reaches its peak; thereafter, the release of ethylene rapidly declines, and by the time the boll undergoes extensive cracking and complete lint emergence, the ethylene release diminishes to its lowest level (Liu et al., 1981).

The maturation, cracking, and lint shedding of cotton bolls are accompanied by dynamic changes in the growth and development of cotton fibers. Cotton fibers originate from specific single-cell protrusions of the ovule epidermis and are formed through a differentiation process. The development cycle of cotton fibers can be roughly divided into four consecutive stages: 1. the initial cell differentiation stage, which signifies the beginning of fiber cell differentiation. 2. the rapid elongation stage, during which the fiber cells undergo rapid elongation; 3. the secondary wall synthesis stage, in which the cell walls thicken and their composition becomes complex and diverse; and 4. the dehydration and maturation stage, during which the fiber cells complete the removal of water, achieving a mature state (Chu, 2023).

**Figure 2:**
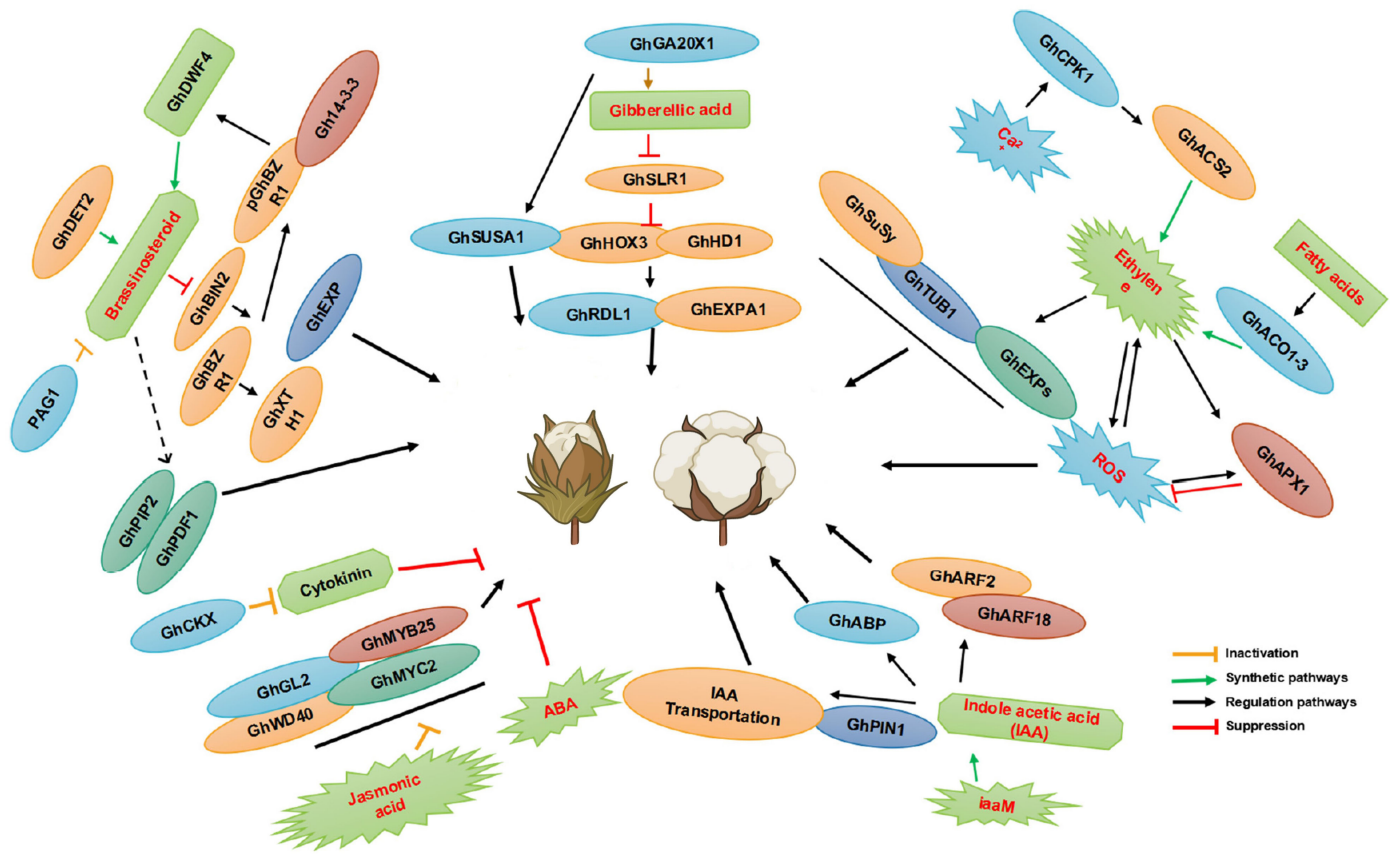
The effects of plant hormones on cotton fiber development. In the diagram, promoting effects are indicated by arrows, whereas inhibitory effects are represented using lines ending with blunt tips. The yellow line indicates the inactivation pathway, the green line indicates the synthesis pathway, the black line indicates the regulation pathway, and the red line indicates the suppression pathway. The figure is modified from reference (Jan et al., 2022).

Plant hormones, naturally occurring small-molecule signaling substances within plants (Rudolf et al., 2024), are indispensable regulatory factors for controlling the growth and development of plants, playing a particularly crucial role in the formation of cotton fiber cells (Daviere and Achard, 2016) (**Figure 2**). Research indicates that hormones such as gibberellic acid (GA), jasmonic acid (JA), auxin, ethylene (ETH), and brassinosteroid (BR) positively promote the development of fiber cells, whereas cytokinins (CK) and ABA exhibit inhibitory effects. Comprehensive studies on endogenous hormone levels have revealed that these hormones not only regulate the initiation and elongation of cotton fiber cells but also significantly influence the retention and abscission of cotton bolls (Ahmed et al., 2020; Mishra et al., 2022).

A quantitative analysis of hormone contents revealed a tight interaction between the initiation and elongation stages of fiber cell development. An analysis using Expressed Sequence Tags (ESTs) derived from ovules showed that hormone regulators such as auxin, BR, GA, and ABA play positive regulatory roles during the early stages of fiber growth, accompanied by the upregulation of specific genes within the *MIXTA*, *MYB5*, *GL2*, auxin, BR, GA, and ETH pathways. However, it is noteworthy that in the n1n1 fiber-deficient mutants, the expression of these key genes is significantly downregulated, directly correlating with the loss of fiber synthesis capability in these mutants. This observation is consistent with previous research on the regulation of immature cotton ovules’ fiber cell development by plant hormones under *in vitro* conditions, demonstrating the intricate regulatory mechanisms of plant hormones in the fiber formation process (Yang et al., 2014; Manghwar et al., 2022).

## 3 Types of chemical harvesting aids

### 3.1 Types of defoliants and ripening agents and their characteristics

Defoliation and ripening agents can be categorized into two primary groups based on their mechanisms of action. The first group comprises contact-type chemicals, including tribufos, dimethipin, carfentrazone-ethyl, glyphosate, paraquat, diuron, and sodium chlorate. These agents inflict direct damage or have lethal effects on the green tissues of plants through various specific mechanisms or biochemical pathways, subsequently triggering the release of ethylene, which facilitates defoliation and ripening. Due to their rapid action, the timing of the application of these compounds in agricultural practices should be relatively late. The second group aims to enhance the production of endogenous ethylene to induce the splitting of cotton bolls and the formation of abscission layers and includes substances such as ethephon and TDZ. In contrast to the first group, the second group of compounds operate in a more gradual and mild manner, necessitating an earlier application in practical agricultural settings to effectively leverage their inducing effects (Chinese Academy of Agricultural Sciences, Cotton Research Institute, 2013). **Table 2** outlines the primary functional characteristics of and considerations for the main defoliation and ripening agents.

**Table 2:** Characteristics of and considerations for main defoliating and ripening agents.

Chemical Components	Functional characteristics	Matters needing attention
Dimethipin	Inhibits protein synthesis, causes rapid water loss from stomata, increases ethylene production, causes leaf drying, and promotes leaf abscission.	Sensitive to alkaline environment, cannot be mixed with pesticides containing heavy metal ions (copper, iron, zinc, etc.).
Tribufos	Destroys the cell layer under the leaf cuticle and stimulates ethylene production, leading to leaf abscission, with no effect on boll or leaf regeneration.	Avoid contact with skin when using.
Ethephon	Promotes cotton leaf abscission and boll maturation and dehiscence, with no effect on leaf regeneration.	Not to be mixed with sodium chlorate, which produces toxic fumes.
Pyraflufen-ethyl	Inhibits the activity of protoporphyrinogen oxidase (PPO) and chlorophyll synthesis, destroys the cell membrane, promotes ethylene production, has good defoliation effect, leads to less leaf adhesion, and has no effect on cotton bolls and leaf regeneration.	Less effective against grass weeds.
Carfentrazone-ethyl		Cannot be mixed with strong alkalis.
Cyclanilide + Ethephon	Promotes boll ripening and cracking and shortens harvesting time.	Less sensitive to temperature, can be mixed with thidiazuron.
Thidiazuron	Increases ethylene production and inhibits growth hormone transport, promoting leaf abscission.	Can be mixed with ethylene.
Paraquat	Stimulates cotton bolls to crack.	Harvest within seven days of application to avoid contamination. The sale and use of paraquat was completely banned in China in 2016.
Sodium chlorate	Promotes shedding of mature leaves.	Inherently unstable and prone to explosion and combustion when mixed with organic substances containing phosphorus, sulfur, etc.
Phosphate-based compounds	Works well to remove the more mature and older leaves from cotton plants.	Has little inhibitory effect on regenerating leaves and is usually mixed with other agents. However, it has an irritating odor of its own.
Glyphosate	May inhibit cotton regrowth and control perennial weeds.	Usually used in combination with defoliant (ethephon).
Diuron	Blocking the light reaction process in plant photosynthesis stimulates plant maturation and leaf abscission.	Toxic—protective measures should be taken when applying.

Based on a comprehensive synthesis of references (Faircloth et al., 2009) and (Edmisten, 2000; Jost and Brown, 2003; Mert, 2007; Sanders et al., 2009).

### 3.2 Types of desiccants and their characteristics

To promote the harvesting of cotton, it is essential that its leaves and bolls remain sufficiently dry. This drying effect occurs either after the plants succumb to biotic and abiotic stresses or through the application of drying agents and other harvest aids before the cotton reaches the maturity and fluffing stages. Aiming to achieve dryness, drying agents work by minimizing the moisture content in leaves and stems during the cotton harvesting process, which facilitates defoliation and boll splitting and fluffing, thereby shortening the cotton’s maturity stage. Harvest aids that result in higher yields and better fiber qualities are classified as drying agents (Boman et al., 2009; Cotton Incorporated, 2017). Drying agents function by directly compromising the structure and function of cell walls, accelerating the rapid loss of intracellular water, and ultimately inducing cell death. From a chemical standpoint, drying agents are categorized as contact-type herbicides, which are intentionally designed to accelerate the drying process of plant tissues and eliminate weeds in cotton fields. This helps remove the obstacles that weeds pose to cotton harvesters during mechanized harvesting and reduces the impurity rate in cotton fibers. Their primary components include, but are not limited to, paraquat, 2,4-dichlorophenoxyacetic acid, glyphosate, and PCP (pentachlorophenol).

Drying agents that are applied during the harvesting process of cotton and other plants are typically associated with hormesis effects. Hormesis is defined as a “biphasic dose-response relationship where a sublethal dose (low dose) induces a beneficial stimulation, and a high dose induces inhibition (toxicity)” (Erofeeva, 2024). Hormesis can also be characterized by the stimulation of various cellular functions with different properties, such as DNA repair, antioxidant defense, and autophagy. Its effects are regulated by the interactions of multiple receptors and signaling pathways, resulting in a comprehensive cellular response (Calabrese et al., 2013). Hormesis represents a coordinated response of organisms to the stresses that they encounter. In plant biology, inducing hormesis can influence cellular and molecular mechanisms in plants, including photosynthesis, the Hill reaction (the process in chloroplasts where water is split under light conditions, releasing oxygen and reducing electron acceptors), chlorophyll content, signaling pathways, and antioxidant enzymes, all of which are closely related to increased crop yields (Mattson, 2008). Additionally, hormesis can enhance crops’ resistance to pathogens and pests (Morkunas et al., 2018).

Reports have documented hormesis effects in barley resulting from the application of acifluorfen-sodium, glyphosate, diquat, haloxyfop-methyl, MCPA (2-Methyl-4-chlorophenoxyacetic acid), metsulfuron-methyl, pendimethalin, and terbuthylazine (Cedergreen, 2008). However, not all herbicides are capable of inducing hormesis effects in plants. The manifestation of hormesis is contingent upon various factors, including the type of herbicide, timing of application, dosage, plant variety, physiological stage, and environmental conditions (Cedergreen and Olesen, 2010).

Lignin enhances the capacity of vascular plants to withstand biotic and abiotic stresses. Conversely, low concentrations of glyphosate significantly decrease the lignin content by inhibiting the shikimate pathway, resulting in improved plant growth (Huang et al., 2024). A reduction in lignin levels in leaves, coupled with an increase in roots, leads to growth retardation and enhanced stress tolerance (Vincent et al., 2005; Yoshimura et al., 2008). The application of low-dose herbicides (2,4-dichlorophenoxyacetic acid, glyphosate, paraquat) modulates plant growth by promoting the synthesis of auxins, activating antioxidant defenses, and facilitating cation transporters in the rhizosphere (Islam et al., 2017). Reactive oxygen species (ROS) serve as signaling molecules that initiate various cellular processes; however, their excessive accumulation can be detrimental to plant tissues (Kumari and Vujanovic, 2020).

The hormetic dose–response relationship represents an evolutionary process in organisms, wherein low-dose stressors (such as chemicals, hormones, and herbicides) activate adaptive mechanisms that enhance plants’ resistance and adaptability to stress, enabling them to withstand a variety of stress challenges (Agathokleous and Calabrese, 2019). Effective hormesis management strategies not only maintain ecological sustainability but also achieve increased agricultural productivity (Calabrese, 2014). Hormesis is one of the most frequently observed phenomena in herbicides with various active ingredients (as shown in **Table 3**). The following sections will introduce several commonly used herbicides.

**Table 3:** Hormesis of different herbicides on different plants at subdoses.

Plant name	Scientific name	Herbicide active ingredient	Dosage (g a. i. ha^-1^)	Effect	References
Rice	*Oryza sativa*	Glyphosate	11.6 – 32.0	Increase the number of grains per spike	(de Castilho Gitti et al., 2011)
Barley	*Hordeum* *vulgare*		11.0 – 45.0	Improvement in photosynthetic efficiency	(Cedergreen and Olesen, 2010)
Barley	*Hordeum* *vulgare*		2.5 – 20.0	Barley production increased by 15 per cent	(Cedergreen et al., 2009)
Chickpea	*Cicer arietinum*		18 - 72	Increase production	(Abbas et al., 2015)
Soya	*Glycine max*		20	30% increase in dry matter weight of seedlings	(Velini et al., 2008)
Corn	*Zea mays*		35	30% increase in dry matter weight of seedlings	
Eucalypt	*Eucalyptus* spp.		7.2	70% increase in total dry matter mass	
Pine	*Pinus caribea*		5.4	20% increase in total dry matter mass	
Corn	*Zea mays*		0.084 and 7.0	16% increase in germination rate	(Barbosa et al., 2017)
Sugarcane	*Saccharum* spp.		1.8	32% increase in leaf number, 32% increase in stems and 6.3% increase in sucrose accumulation	(Pincelli-Souza et al., 2020)
Sugarcane and Eucalypt	*Saccharum* spp. and *Eucalyptus* spp.		6.2 - 20.2	The total dry matter weight of sugarcane and eucalyptus increased by 28.8% and 35.5%, respectively, and the transpiration rate of sugarcane increased by 80.6% and that of eucalyptus by 86.1%.	(Nascentes et al., 2018)
Sugarcane	*Saccharum* spp.		72 - 180	Increased phenylalanine and tyrosine levels and plant stem biomass	(Carbonari et al., 2014)
Parica(A tree species found in tropical rainforests of South America)	*Schizolobium amazonicum*		9,18,36	39.3% increase in plant height	(Marques et al., 2020)
Coffee	*Coffea arabica*		416 - 738	Plant height, stem thickness, number of leaves, leaf area and dry matter all increased by 18%-39%	(Carvalho et al., 2013)
Kidney bean	*Phaseolus vulgaris*		12	Increases the nutrient content of beans and promotes higher yields up to 375 kg/ha	(Silva et al., 2016)
Tomato	*Solanum lycopersicum*		0.2-5	Promotes hypocotyl and radicle growth and can increase photosynthetic rate twofold	(Khan et al., 2020)
Wheat	*Triticum aestivum*		18	Stimulates growth and increases yield	(Abbas et al., 2016a)
Corn	*Zea mays*		36	80% increase in leaf dry matter and 19% increase in plant above-ground dry matter	(Latorre et al., 2010)
Corn	*Zea mays*		3.5	10% and 16% increase in germination rate	(Barbosa et al., 2017)
Upland cotton	*Gossypium hirsutum*		20	Promotes 9-13% increase in cotton plant height, increase in number of bolls per plant, and increase in fiber maturity and macronutrient value	(Ferrari, 2015)
Kidney bean	*Phaseolus vulgaris* L		10	Increase production	(da Silva et al., 2016)
Eucalyptus grandis	*Eucalyptus grandis*		1.9 - 3.7	Promotes root elongation and dry matter increase	(Velini et al., 2008)
Pinus caribaea	*Pinus caribea*		2.0 - 14.6	Stimulates plant growth with 52% increase in root dry matter	(Velini et al., 2008)
Barley	*Hordeum* *vulgare*	Glyphosate and metsulfuron-methyl	143 and 1.3	25% increase in biomass accumulation	(Cedergreen, 2008)
Soybean	*Glycine max*	2,4-D	19.2 - 20.3	Increase the number of grains per plant and grain weight	(Silva et al., 2019)
Cotton	*Gossypium hirsutum*		1.75	Increase production	(Americo et al., 2017)
Brazilian oil peach wood	*Caryocar brasiliense*		3.3	20.7% increase in leaf area	(Tavares et al., 2017)
Wheat	*Triticum aestivum*		5.0-20.0	Increased chlorophyll content index and chlorophyll fluorescence value	(Kaur and Kaur, 2019)
Upland cotton	*Gossypium hirsutum*		0.855 - 1.71	Promote increase in plant height, leaf number, branch dry matter, and total dry matter	(Marques et al., 2019)
Citrus	*Citrus reticulata*		10.0 mg/L	Increase acidity and decrease soluble solids/fruit acidity ratio	(Rufini et al., 2008)
Citrus	*Citrus reticulata*		50 mg/L and 100 mg/L	Increased fruit size, peel weight and juice content	(El-Otmani et al., 1993)
Upland cotton	*Gossypium hirsutum*		2.72	Promoting a 21% increase in cotton production	(Furlani et al., 2011)
Upland cotton	*Gossypium hirsutum*		0.68 - 1.9	Increased cotton seed harvest	(Am´erico et al., 2016)
Tomato	*Solanum lycopersicum*		6.6	Promotes early ripening of tomatoes and increases yields by 6.3%-14.03%	(Hemphill and Montgomery, 1981)
Potato	*Solanum tuberosum*		16	Increased production	
Cucumber	*Cucumis sativus*		110	Increased production	
Carrot	*Daucus carota*		44	Increased production	
Eucalyptus grandis	*Eucalyptus grandis*		0.94 - 3.75	12% increase in plant height and 11% increase in stem diameter	(Fragoso, 2014)
Brazilian oil peach wood	*Caryocar brasiliense*		3.3	20.7% increase in leaf area, 27.1% increase in stem dry weight, 32.45% increase in root dry weight, 46.7% increase in leaf dry weight	(Tavares et al., 2017)
Soya	*Glycine max*	Chlorimuron-ethyl and 2,4-D	0.4 and 20.0	Promoted plant height, stem diameter, biomass accumulation and dry matter increase	(Silva et al., 2020)
Rye and Peas	*Secale cereale* and *Pisum sativum*	Simazine	0.05 and 0.8 μM	79% increase in protein content and increase in nitrate reductase activity	(Ries et al., 1967)
Soya	*Glycine max*	Sulfentrazone and lactofen	9.0 and 70.0	Increased production of plant antitoxins and reduced lesions caused by *Dictyostelium nucleatum*	(Nelson et al., 2002)
Upland cotton	*Gossypium hirsutum*	Paraquat	4.8 - 24	Cotton seed receipts increased by 29.6%	(Melero, 2016)

Organized according to references (Jalal et al., 2021; Mielke et al., 2022).

#### 3.2.1 Glyphosate

Glyphosate is the most widely used non-selective herbicide globally. At the recommended application rates, it inhibits the enzyme 5-enolpyruvylshikimate-3-phosphate synthase (EPSP synthase), the synthesis of aromatic amino acids (such as tryptophan, tyrosine, and phenylalanine), and photosynthesis. Additionally, it upregulates related genes, leading to the accumulation of glutathione and homoglutathione, ultimately causing plant death (Vivancos et al., 2011). Conversely, at low doses, glyphosate can stimulate the accumulation of shikimate, enhance photosynthesis and stomatal conductance, shorten the plant’s lifecycle, promote growth, and increase the yield potential (Brito et al., 2017).

The inhibition of EPSP synthase also impacts the shikimate metabolic pathway, which is essential for lignin synthesis. Low doses of glyphosate can effectively suppress lignin formation, maintaining cell wall elasticity for prolonged periods and thus allowing for greater cellular expansion and growth (Meschede et al., 2011). In sugarcane cultivation, glyphosate is extensively used as an herbicide for weed control at application rates ranging from 64.8 to 777.6 g a.i. ha^-1^. Sublethal doses of glyphosate inhibit lignin synthesis, permitting assimilated carbon that is not allocated to lignin formation to be utilized for increasing the sucrose content (Dusky et al., 1985). The application of glyphosate at 1.8 g a.i. ha^-1^ has been shown to stimulate sugarcane’s primary growth, dry matter accumulation, productivity, sucrose content, and phosphorus uptake and transport (Pincelli-Souza et al., 2020). Furthermore, when applied at rates of 108-216 g a.i. ha^-1^, glyphosate can also serve as a ripening agent (de Araujo, 2015). Lower doses of glyphosate may induce hormesis effects by promoting plant growth and altering the morphological structures of plants. The application of low-dose glyphosate at 3.5 g a.i. ha^-1^ can stimulate the growth and development of maize, resulting in a 10% increase in germination rate and a 16% improvement in physiological performance (Barbosa et al., 2017).

#### 3.2.2 Paraquat

Paraquat is a non-selective, contact-type herbicide that rapidly impacts the photosynthetic system of plants (Ekmekci and Terzioglu, 2005). It interacts with O_2_ to inhibit the reduction of NADP^+^ to NADPH_2_ in photosystem I, leading to the excessive generation of superoxide radicals (O_2_^-^) and reactive oxygen species (ROS), which negatively affect plant growth. However, at low concentrations, paraquat can enhance plants’ antioxidant defense capabilities and decrease their ROS production (Somboon et al., 2019).

#### 3.2.3 Fenoxaprop-P-ethyl

Fenoxaprop-P-ethyl is a selective, systemic, translocated herbicide that is effective against monocotyledonous weeds. At the recommended doses, it inhibits acetyl-CoA carboxylase in weeds, thereby suppressing fatty acid synthesis, halting the growth of meristematic tissues, and causing stem and leaf wilting. Low doses of fenoxaprop-P-ethyl exhibit hormesis effects, promoting branch growth by up to 39% (Petersen et al., 2008). The hormesis dosage range for fenoxaprop-P-ethyl is 1 - 6 g a.i. ha^-1^, which can enhance the growth of oats and canary grass (Abbas et al., 2016b). The application of fenoxaprop-P-ethyl at 2%-20% of the recommended dosage induces hormesis effects in Phalaris minor Retz (Farooq et al., 2019).

#### 3.2.4 2,4-Dichlorophenoxyacetic acid

2,4-D is a selective herbicide based on auxin and is also recognized as a growth regulator that can enhance plant growth at lower concentrations. The hormesis phenomenon associated with 2,4-D is linked to the production of auxin (Tavares et al., 2017), and promotes plant growth by enhancing nucleic acid metabolism, cell wall plasticity, cell elongation, and division. Additionally, 2,4-D can regulate the maturation of citrus fruits, thereby reducing fruit drop or extending the harvest period. The combined application of 25 mg/L of gibberellic acid (GA3) and 25 mg/L of 2,4-D can decrease the natural fruit drop rate in citrus by 78% and suppress fruit drop for up to three months (Almeida et al., 2004). In comparison to untreated controls, treatment with 16 mg/L of the isopropyl ester form of 2,4-D can reduce citrus fruit drop by 62% (Anthony and Coggins, 1999).

Sublethal doses of 2,4-D promote the morphological characteristics, dry matter accumulation, and yield traits of cotton plants. Spraying 0.855 and 1.71 g a. i. ha^-^¹ of 2,4-D choline salt spray during the cotton’s B4 stage (during which each plant has four young bolls) can increase the above-ground and total dry matter content, number of leaves, and plant height (Marques et al., 2019). Additionally, it has been reported that sublethal doses of 2,4-D ranging from 1.90 to 2.72 g a. i. ha^-^¹ can enhance cotton yield compared to higher doses (Americo et al., 2017). Furthermore, applying 2,4-D at concentrations of 5-20 g a. i. ha^-^¹ can increase chlorophyll content and chlorophyll fluorescence parameters in wheat leaves (Kaur and Kaur, 2019).

However, it is important to note that the application of the aforementioned herbicides may cause plants or weeds to develop herbicide resistance, making it difficult to achieve the intended defoliant maturation purpose. The development of resistance mainly arises from naturally occurring genetic variations within weed populations, which enable some weed individuals to tolerate herbicide doses that would normally kill most wild-type weeds. Herbicides themselves do not directly cause the emergence of resistance but act as selective pressure, selecting for resistant weed individuals through repeated use. When weed populations are frequently exposed to specific herbicides or herbicide combinations, individuals with genetic resistance can survive and reproduce, gradually increasing their gene frequency within the population, ultimately leading to enhanced resistance of the entire weed population to that herbicide or herbicide combination. Therefore, during use, it is necessary to prevent the sole application of or over-reliance on herbicides (in combination with manual weeding, tillage, crop rotation, etc.) to avoid the formation and development of herbicide resistance patterns in weeds. Additionally, applying sublethal doses of herbicides or failing to apply herbicides in a timely manner may also promote the development of resistance (Gaines et al., 2020).

## 4 Factors affecting the effectiveness of defoliation and ripening

Beyond the selection of harvest aid varieties, as well as the proportions and methods of their mixed application, the intrinsic growth and developmental status of the plants also influences the actual efficacy of harvest aids. Temperature and moisture are critical factors in the defoliant-induced maturation process. Compact and dense cotton plants are more favorable for the timely maturation of bolls and subsequent leaf abscission (Wright et al., 2015). Achieving these conditions can be facilitated through the use of plant growth regulators or agronomic management practices, such as the application of mepiquat chloride for topping, optimal sowing dates, appropriate plant density, and nitrogen fertilizer application. Depending on the plants’ growth and developmental status, variations in temperature and humidity, water availability, as well as differences in the types and ratios of chemical harvest aids, leaves begin to abscise 2-7 days after defoliant application, with complete leaf abscission typically requiring 10-14 days (Stewart et al., 2000).

In summary, the factors influencing the effectiveness of cotton defoliant-induced maturation can be categorized into two groups: internal factors, which include the crop’s inherent growth and developmental status (such as maturity and canopy structure), and external factors, encompassing environmental conditions, pests and diseases, as well as other biological and abiotic stresses.

### 4.1 Stage of growth

To achieve better defoliation results, the growth and development status of cotton plants in the field should be kept relatively consistent. Before applying defoliants, it is advisable to use plant growth regulators such as mepiquat chloride and chlormequat to shorten internode length, suppress vegetative growth, maintain a compact plant structure, reduce boll shedding, and increase boll retention rates. The growth vigor of cotton populations depends not only on soil fertility and environmental conditions but also closely on their varietal characteristics. Long-branch genotypes are more prone to defoliation compared with short-branched genotypes. Harvest aids and similar substances exhibit better defoliant efficacy in genotypes with sparse and deeply lobed leaves, whereas they perform poorly in genotypes with dense and large leaves. Under conditions of excessive nitrogen application, cotton leaves show reduced sensitivity to defoliants, resulting in less leaf abscission (Feng et al., 2017).

Furthermore, the balance between vegetative growth and reproductive growth during the entire growth period of cotton plants is a critical factor affecting their response to defoliant-induced maturation agents. Environmental factors or agronomic practices that favor vegetative growth over reproductive growth can diminish the effectiveness of defoliants, leading to reduced cotton yields and an inferior fiber quality (Gormus et al., 2017). Research has shown that applying defoliants when the boll shedding rate is between 76.5% and 89.0% can significantly enhance the lint cotton yield. In contrast, when the boll shedding rate ranges from 40% to 60%, the application of defoliants results in the optimal cotton fiber quality. This indicates that the peak yield and optimal fiber quality do not occur at the same stage of crop maturity, which may correspond to the biological process in which fiber elongation precedes fiber filling (yield) (Bednarz et al., 2002).

During the initial vegetative growth phase of cotton, seedlings first develop two cotyledons, followed by significant internodal elongation, resulting in the formation of five to nine primary stem nodes and their corresponding leaves. Subsequently, cotton transitions into its reproductive growth stage, during which lateral buds in the leaf axils of the main stem progressively differentiate into reproductive branches, a process that typically initiates at the fifth to ninth nodes of the main stem. As the nodes on the main stem continue to develop, they transform into fruiting branches (FBs), with most subsequent nodes generating fruiting branches in sequence. It is important to note that the continuous flowering of cotton relies on the sustained supply of vegetative growth, which, through processes such as photosynthesis, generates assimilates that provide the necessary material foundation for the formation and development of additional fruiting branch nodes (Brecke et al., 2001).

Cotton plants display a characteristic of continuous growth, with both its vegetative and reproductive growth processes being able to proceed sustainably under suitable environmental conditions and an adequate nutrient supply. When reproductive growth advances smoothly and the first cotton boll (i.e., the fruit that is closest to the main stem on the fruiting branch) successfully forms, the bolls act as significant reservoirs of carbohydrates, thereby suppressing the rate of vegetative growth in the later stages of the plant. Throughout the entire growth cycle, the early formation and retention of the first boll effectively facilitate the redistribution of carbohydrates from vegetative branches and root growth to the development of bolls (particularly cotton fibers and seeds). If the first boll is shed or its development is impeded, the plant will continue to grow until natural senescence occurs at the end of the season. Therefore, in cotton production practices, precise regulation is required to achieve a dynamic balance between vegetative and reproductive growth to maximize the harvest efficiency. This involves promoting early flowering and fruiting and ensuring the retention of the first boll throughout the growth period, thereby aligning reproductive maturation with effective boll setting. Furthermore, the vegetative nodes of cotton plants contribute relatively little directly to the total yield, whereas the reproductive nodes that are derived from fruiting branches form the main body of cotton yield. The exclusive use of chemicals such as ethephon may reduce the number of reproductive nodes, thereby diminishing the cotton yield potential, a possibility that should receive full attention in production management (Da Costa and Cothren, 2011).

Nodes above the white flower (NAWFs) are defined as the number of main stem nodes or fruit branches that can be counted sequentially from the first node bearing a white flower at the uppermost part of the cotton plant. NAWFs can be utilized to assess the maturity of cotton, with NAWFs=5 signifying physiological maturity (Dong et al., 2005).

Additionally, research has shown that monitoring the height-to-node ratio (HNR) can be used to evaluate the vegetative growth status of cotton plants. The HNR is calculated by dividing the plants’ height by the number of main stem nodes, resulting in the average internodal length. When the average internodal length surpasses a specific value for a given developmental stage (**Table 4**), the cotton plant is classified as undergoing vegetative growth (Jost et al., 2005).

**Table 4:** HNR values of cotton during different growth stages.

Growth stage	Growth stage		
	Normal	Stressed	Overnutrition
Seedling	0.5-0.75	–	–
Early squaring	0.75-1.2	0.7	>1.3
Large square–1st bloom	1.2-1.7	<1.2	>1.9
Early bloom	1.7-2.0	<1.6	>2.5
Early bloom + 2 weeks	2.0-2.2	<1.8	>2.5

Monitoring the growth rate of cotton from the 8- to the 10-leaf stage onwards is one of the key indicators for assessing its growth vigor. Considering the variability in planting row spacing and cultivation practices, it is recommended to use the vertical distance from the base of the cotyledons (i.e., the first pair of cotyledons that are directly attached to the main stem at the time of emergence) to the apical growth point (i.e., the terminal bud) as the measurement standard, rather than the direct vertical height from the ground to the apex. In this measurement method, the cotyledon attachment point is designated as node 0. As the plant develops, the cotyledons are shed, forming two small nodes near the base. Subsequently, the first true leaf (i.e., node 1) emerges at the apex within 7 to 10 days post-emergence. Following this, the leaves on the main stem appear sequentially at intervals of approximately 3 days (or extended to 4 days under adverse environmental conditions), with each new leaf occupying an independent node, and the stem segments between nodes being referred to as internodes (Wang et al., 2024).

Fruiting branches typically initiate development within the fifth to seventh node interval of the main stem, arising from the differentiation of lateral buds in the leaf axils or side buds at the base of the main stem. These fruiting branches periodically form reproductive buds or shoots with opposite leaves at different nodes along the branches at intervals of approximately 6 days (which may extend to 7 to 9 days under environmental stress), with designations such as FB1 representing the first position. These structures serve as the primary sources of assimilates for buds (which bloom approximately 21 days later) and subsequent cotton bolls (which mature within approximately 6 weeks from flowering). Additionally, vegetative branches (averaging two to three per plant) may develop from nodes below the first fruiting branch on the main stem, adjacent main stem leaves, or from second lateral buds when the fruiting branches are damaged. In the early stages of management, maintaining a retention rate of no less than 80% for buds at the FB1 position is regarded as one of the key measures for optimizing the cotton yield potential (Sun et al., 2016).

### 4.2 Temperature

Temperature, as a key factor regulating the defoliation process, significantly affects the functional characteristics of cotton leaves, which are critical organs. Under extreme drought and high-temperature conditions, cotton leaves adapt to the environment by developing a thickened wax layer as a protective mechanism, effectively reducing the loss of water through evaporation. However, this wax barrier simultaneously impedes the effective penetration and absorption of exogenous chemical substances such as harvest aids. In contrast, under mild and suitable temperature conditions, the thickness of the leaf wax layer tends to decrease, and its structure transforms into a more flexible, spongy form. This change facilitates the rapid penetration and efficient absorption of harvest aids on the leaf surface, thereby promoting the subsequent defoliation process (Tian et al., 2004).

The field temperature is a critical factor influencing the efficiency of cotton defoliation. The optimal temperature for defoliant application is approximately 25°C. At low temperatures, cotton’s metabolism slows, leading to reduced absorption efficiency of defoliants. Additionally, low temperatures suppress the synthesis of ethylene and auxin in the AZ and the response of their signaling pathways to TDZ. This suppression prevents the expression of cell wall hydrolase genes, thereby inhibiting the separation of AZ cells and the abscission of cotton leaves, resulting in green leaves that do not fall (Shu et al., 2022). Conversely, at high temperatures, cotton’s metabolism accelerates, causing excessive absorption of defoliants. This leads to scorched or wilted cotton leaves that do not abscise (Feng et al., 2021).

Cotton’s growth and development can be monitored using HUs (heat units), calculated as follows: (maximum temperature + minimum temperature)/2 - 15.5°C. An HU is defined as the accumulated heat within 24 hours when the average ambient temperature exceeds the base threshold temperature of 15.5°C. Studies have shown that combining the cumulative HU after NAWF=5 with traditional methods can verify the appropriate timing for defoliant application. Applying defoliants at NAWF=5 and HU=472 can significantly increase the yield of lint cotton, but the HU value may vary considerably due to differences in environmental conditions across different regions (Gwathmey et al., 2004).

Research has indicated that the growth and development of cotton are generally modeled using a heat unit system based on a base temperature of 60°F (Fahrenheit scale, 1°C=33.8°F) (**Table 5**). In this model, the heat unit is referred to as DD-60s, which is calculated by subtracting 60°F from the daily average temperature using the following formula: [Max°F (maximum temperature) + Min°F (minimum temperature)]/2 - 60°F. Since cotton plants may not grow at temperatures above 93°F, any temperature exceeding 93°F should be capped at 93°F in the calculations (Ritchie et al., 2007).

**Table 5:** Accumulation of DD-60s during different growth stages of cotton.

From planting to	Required temperature/DD-60s	Required time/d
Emergence	50	4-14
Pinhead square	550	35-45
First bloom	940	55-70
Peak bloom	1700	85-95
First open boll	2150	115-120
Harvest	2500-2700	140-160

### 4.3 Plant diseases and insect pests

Verticillium wilt, as a fungal disease that infects cotton, involves a pathological mechanism that includes the blockage of the plant’s xylem, disrupting the normal transport of water and nutrients and weakening the efficacy of chemical aids that are applied during the harvest period. In the early stages, the disease manifests as a gradual wilting of cotton leaves, and as it progresses, it induces the shedding of leaves and cotton bolls. Moreover, if the disease infects cotton mildly towards the end of the growing season, it stimulates excessive production of ethylene. Ethylene, as a plant hormone, promotes the formation of the abscission layer, ultimately accelerating the leaf abscission process and adversely affecting the yield and quality of the cotton (Zhu et al., 2023).

Target spot, a foliar disease caused by the fungal pathogen *Corynespora cassiicola*, affects crops’ leaves. Its characteristic symptoms initially present as brick-red spots on the leaves, forming irregular or circular lesions with a brownish or light brown center. Under conditions of sustained leaf wetness, the disease spreads rapidly, typically starting from the lower leaves of the plant and progressively moving upward. As the infection intensifies, it can result in up to 70% of the leaves shedding prematurely, and both the cotton boll bracts and the bolls themselves may become infected (Fulmer et al., 2012). Diseases such as target spot can impair the functionality of cotton leaves, reduce their photosynthetic efficiency, limit the nutrient supply that the plant and cotton bolls need, cause premature leaf damage and shedding, and, in severe cases, lead to poor development or the abscission of cotton bolls, thereby exacerbating yield losses (Rondon M and Lawrence, 2021).

Insect feeding activities pose a significant threat to cotton, not only directly causing leaf deformation and abscission but also exacerbating boll shedding rates and potentially impairing the seed quality and cotton fiber characteristics. The causes of boll shedding are complex and varied, as it can be directly attributed to insects feeding on bolls or indirectly to insects extracting nutrients from leaves, petioles, or stems. The latter exacerbates the shedding phenomenon by reducing the effective leaf area and inducing physiological disorders in the plant (Luttrell et al., 2015). For example, the larvae of pests such as the cotton bollworm (Pectinophora gossypiella Saunders), the cotton bollworm (Helicoverpa zea Boddie), and the tobacco budworm (Heliothis virescens Fabricius) feed extensively on the leaves, buds, flowers, and young bolls of cotton, not only causing direct tissue damage but also indirectly stimulating ethylene biosynthesis, thereby triggering abscission (Wu and Guo, 2005). In the later stages of cotton plants’ growth and development, the application of harvest aids can prevent the reproduction of these pest populations on cotton leaves. Defoliation of the lower canopy allows for improved ventilation and light penetration in the lower parts of the plant, reduces the relative humidity, and decreases the incidence of pests such as *Bemisia tabacii Gemi*. (whitefly) and *Aphis* spp. (aphids), as well as diseases such as *Ascohyta* spp., *Glomerella* spp., and *Alternaria* spp., which cause boll rot (Oğlakçı et al., 2007).

The severity of pest and disease attacks and their timing within the growing season can induce abnormal growth or an imbalance in vegetative growth, promote early boll abortion, and ultimately delay the overall maturation process of cotton plants. In view of this, implementing effective pest and disease control management strategies is of critical importance for maintaining the balance between vegetative and reproductive growth, ensuring the timely maturation of plants, and laying a solid foundation for successful harvesting. Additionally, interruptions in boll setting that are caused by pest and disease attacks can lead to uneven boll development, not only increasing the complexity of the harvesting management process but also affecting the precision of the timing of harvest aid application. Pest and disease attacks permeate the entire cotton growth process, but damage occurring during the bud and flower boll stages is particularly crucial. This may disrupt the normal maturation and senescence trajectory of cotton plants through boll shedding and subsequent compensatory growth mechanisms, inducing excessive vegetative growth and thereby significantly negatively impacting the efficacy of harvest aids. Therefore, before the application of harvest aids, preventing cotton plants from being afflicted by pest and disease attacks and maintaining the overall health of leaves and plants are of significant importance for ensuring a good cotton yield.

### 4.4 Maturity of cotton bolls

**Figure 3:**
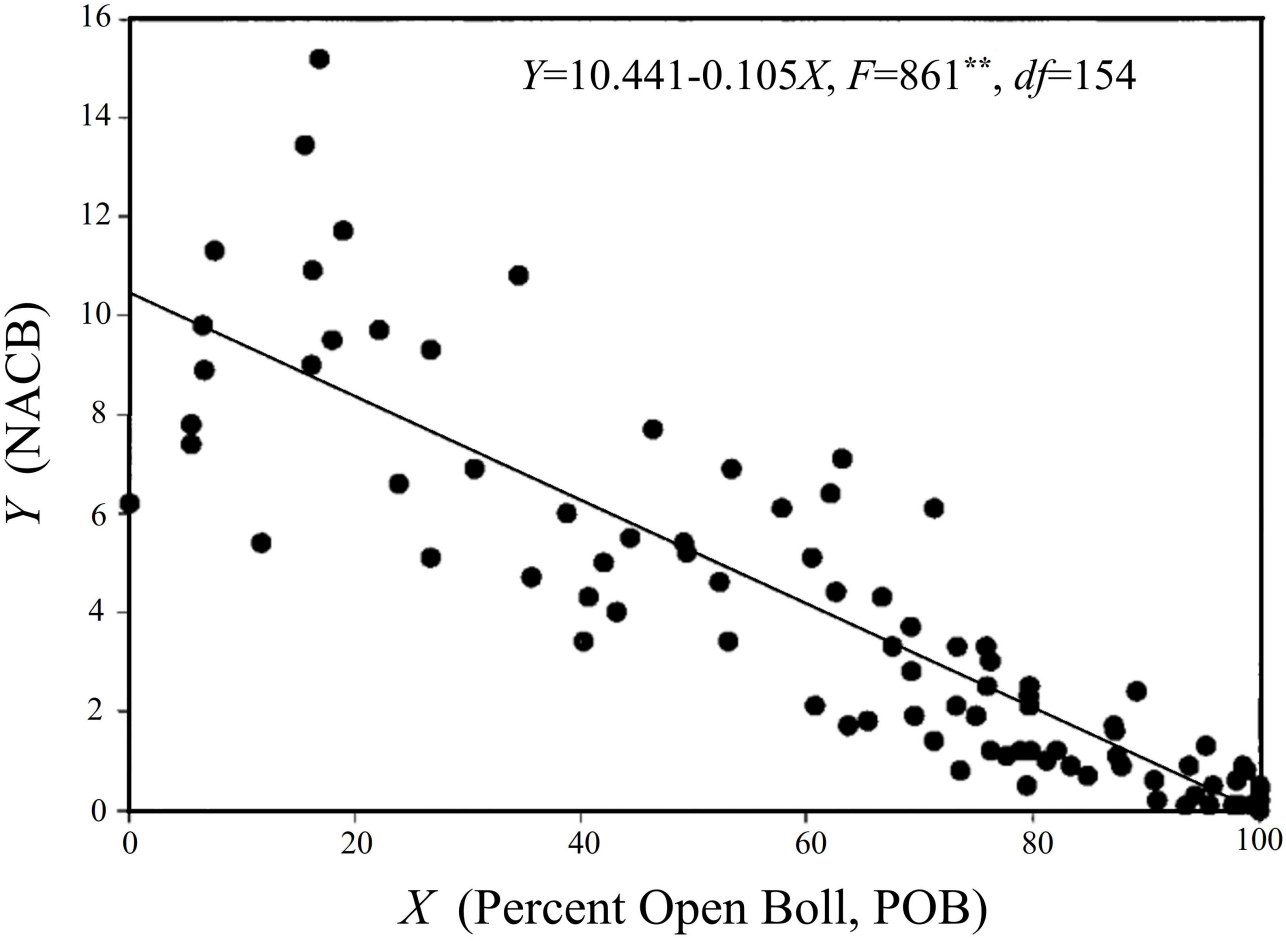
Relationship between NACB and POB. The figure is modified from reference (Bednarz et al., 2002).

The primary objective of applying defoliant harvest aids is to expedite the maturation and cracking of cotton bolls. Consequently, selecting suitable defoliant harvest aids based on the developmental maturity of the cotton bolls is a crucial measure to ensure both a good cotton yield and fiber quality. A percent open boll (POB) value reaching 60%, as well as a visual maturity assessment exceeding 90% (in terms of seed coat and fiber development) after cutting open the bolls can both serve as indicators to determine the timing of defoliant application. Furthermore, the time at which the number of nodes above the cracked boll (NACB) is equal to four (**Figure 3**) is designated as the appropriate time for defoliant application. At this point, defoliation of the cotton plants will not negatively impact the yield or fiber quality (Supak and Snipes, 2001).

Additionally, the maturity of cotton bolls can be assessed based on their size, with “suitable for harvest cotton bolls” being defined as individuals with a diameter exceeding 2 cm and exhibiting an expanded and closed state (Gwathmey et al., 2016). Although some late-maturing bolls may not meet the maturity criteria before the application of harvest aids, if they possess physiological maturity potential and grow under favorable meteorological conditions post-application, most of these bolls are expected to complete the maturation process and crack open smoothly, thereby increasing the proportion of harvestable bolls (Faircloth et al., 2004).

The maturation process of cotton bolls involves two main stages: physiological maturity and fiber maturity. Physiological maturity marks the complete maturation of seeds and fibers within the cotton boll, while fiber maturity, which is essential for efficient harvesting, refers specifically to the stage where cellulose deposition in the secondary cell wall is finalized (Bruns, 2009). When cotton bolls achieve a mature state consisting of both seeds and fibers, it indicates that they have reached a suitable stage for the application of harvest aids in preparation for harvesting. At this juncture, the application of harvest aids does not adversely affect the quality of the cotton fiber, and the crop’s growth age and physiological state synergistically enhance its response to harvest aids. Compared with cotton with lower maturity levels, naturally senescing cotton exhibits a more sensitive response to harvest aids, particularly under conditions of heavy boll loads (Brecke et al., 2001). Therefore, with the appropriate management and favorable climatic conditions, the application of harvest aids will facilitate the smooth cracking of physiologically mature cotton bolls, thereby releasing mature fibers for harvest.

### 4.5 Moisture content

In cotton cultivation, maintaining suitable soil moisture conditions is essential to ensure that the cotton bolls receive an adequate water supply throughout their three- to four-week maturation period. Under water stress, the total dry matter accumulation in cotton plants decreases, the leaf water potential (ψWL) declines, stomata close, and photosynthesis is reduced. During reproductive development, water stress can lead to higher boll shedding rates and a decreased cotton yield (Pettigrew, 2004). Drought stress results in cotton leaves developing a thicker cuticle, which inhibits the absorption of defoliants, thereby diminishing the efficacy of defoliant-induced maturation. Excessive moisture promotes vigorous vegetative growth, resulting in dense foliage and canopy shading. In most cases, higher application rates increase the risk of leaf adhesion or death; thus, it is not advisable to increase the dosage of harvest aids. To ensure complete pesticide coverage, a second application can be carried out after the initial spray around the canopy periphery and subsequent leaf fall (Sanders et al., 2009).

To predict the crop status following the final irrigation, it is essential to consider multiple factors comprehensively, including the meteorological conditions, soil’s water-holding capacity (which is affected by the soil texture), irrigation volume, boll load, and overall condition of the canopy. Typically, to optimize late-season crop management, the interval between the last irrigation and the application of chemical harvest aids should be extended to twice the standard late-season irrigation interval. This strategy aims to first meet the basic water needs for late-season boll maturation and subsequently promote the natural senescence of the crop through moderate drying. When applying the “double interval time for late-season irrigation” empirical rule, it is crucial to accurately evaluate the consumption of plant-available water (PAW) in the soil after irrigation. This involves understanding the volume of the previous irrigation, the textural characteristics of the deep soil (the effective root distribution zone), and the depth of the soil profile when saturated with irrigation water. Then, by regularly monitoring and deducting the estimated weekly evapotranspiration (ET), the rate of plant-available water depletion in the soil can be effectively tracked and predicted, providing a scientific foundation for precise irrigation management (Silvertooth, 2001).

Although cotton is regarded as a crop with a certain level of drought tolerance, its normal growth and development remain highly dependent on an adequate water supply. When cotton leaves transition from bright green to dark green, accompanied by wilting, and the stem color changes from light green to red, it indicates that the cotton plants are undergoing water stress. At this stage, implementing remedial irrigation measures can mitigate the stress condition, but it remains challenging to completely eliminate the negative effects on the final yield. The water requirements from bud initiation to post-flowering in cotton are particularly crucial, directly influencing the yield. Therefore, it is recommended that after the first flowering of cotton, precise measurement techniques be utilized to ensure that the total water demand for this stage—as detailed in the table below—is satisfied through a combination of natural rainfall and artificial irrigation, thereby optimizing the yield potential (**Table 6**) (Kemerait, 2021).

**Table 6:** Recommended water requirements under high cotton yields.

Stage	Centimeters/Week	Centimeters/Day
Week beginning at 1^st^ bloom	2.54	0.381
2^nd^ week after 1^st^ bloom	3.81	0.5588
3^rd^ week after 1^st^ bloom	5.08	0.762
4^th^ week after 1^st^ bloom	5.08	0.762
5^th^ week after 1^st^ bloom	3.81	0.5588
6^th^ week after 1^st^ bloom	3.81	0.5588
7^th^ week and beyond	2.54	0.381

This table is modified from reference (Kemerait, 2021).

The University of Georgia (UGA), through 15 years of comprehensive research in the region, has developed an innovative irrigation management strategy called the “UGA Checkbook”, which is tailored to the average evapotranspiration rates of local cotton cultivation areas. This approach, grounded in thorough data analysis, establishes scientific guidelines for the water needs across the entire growth cycle of cotton. It delineates recommended standards for the combined total of natural rainfall and supplemental irrigation to ensure that the cotton receives sufficient water, thereby supporting its normal growth and development (**Table 7**) (Hand et al., 2023).

**Table 7:** Water requirements for the entire growth period of cotton.

Growth stage	Days after planting	Weeks after planting	Water demand	
			Centimeters/Week	Centimeters/Day
Emergence	1-7	1	0.1016	0.0254
Emergence to first square	8-14	2	0.4572	0.0762
	15-21	3	0.7366	0.1016
	22-28	4	1.0414	0.1524
	29-35	5	1.4224	0.2032
First square to first flower	36-42	6	1.8034	0.254
	43-49	7	2.159	0.3048
	50-56	8	2.7432	0.381
First flower to first open boll	57-63	9	3.2512	0.4572
	64-70	10	3.7338	0.5334
	71-77	11	3.8608	0.5588
	78-84	12	3.7592	0.508
	85-91	13	3.6068	0.508
	92-98	14	3.302	0.4826
	99-105	15	2.9464	0.4318
	106-112	16	2.2352	0.3302
	113-119	17	1.7526	0.254
First open boll to >60% open bolls	120-126	18	1.2954	0.1778
	127-133	19	0.889	0.127
	134-140	20	0.5588	0.0762
	141-147	21	0.3048	0.0508
	148-154	22	0.127	0.0254
	155-161	23	0.0508	0
Harvest	162-168	24	0	0
	169-175	25	0	0

This table is modified from reference (Hand et al., 2023).

### 4.6 Other influencing factors

Nitrogen (N) is a critical macronutrient that plays an indispensable role in the growth and development of cotton. As a constituent of chlorophyll, nucleic acids, membrane proteins, enzymes, and plant hormones, nitrogen regulates fundamental physiological processes, including photosynthesis, cellular division, and overall metabolic function. While an adequate nitrogen supply is essential for optimizing fruit-setting patterns, enhancing boll retention rates, and ensuring timely maturation of cotton, an excess of nitrogen can hinder the efficacy of harvest aids. Excessive nitrogen primarily promotes vigorous vegetative growth, which shifts the distribution of photosynthates towards vegetative organs at the expense of reproductive tissues. This imbalance disrupts reproductive development, leading to delayed boll maturation, lower boll retention, and potential premature abscission of immature bolls. Furthermore, nitrogen enrichment can result in abnormal leaf enlargement, a process that is influenced by both the nitrogen and water availability, potentially exacerbating the effects of poor boll maturation by obstructing lower bolls and delaying the abscission process (Kerby et al., 1987; Jackson and Gerik, 1990).

When harvest aids are applied, if the nitrogen has not been fully utilized by the plant and the moisture levels remain sufficient, the cotton plant may continue to exhibit healthy, vigorous growth. This late-season excessive growth prevents the plant from reaching the necessary senescence threshold for effective leaf abscission. Consequently, the failure to induce sufficient leaf senescence significantly impairs the efficacy of defoliants, which is a critical issue for crop management in the late growing season (Cathey, 1986). Brown (Brown and Rhyne, 1954) demonstrated that the efficiency of leaf abscission is positively correlated with leaf age, highlighting that older leaves, especially those located beneath mature bolls, are more responsive to defoliants. In contrast, younger leaves, particularly newly emerged ones, display greater resistance to defoliants and fail to abscise efficiently, further complicating the defoliation process (Thomas, 1965).

Beyond nitrogen, other macronutrients such as phosphorus (P) and potassium (K) are pivotal in determining the overall health and productivity of cotton. Phosphorus is crucial for energy transfer and storage within the plant, influencing processes such as root development, early plant growth, and flower induction. Adequate phosphorus levels promote efficient nutrient uptake and are necessary for proper boll formation and fiber quality, thereby indirectly enhancing the plant’s response to harvest aids. However, insufficient phosphorus can hinder root function and delay reproductive development, which may also interfere with the timely application of harvest aids (Girma et al., 2007). Potassium, on the other hand, is integral to the regulation of water balance and stress tolerance in cotton. Potassium deficiencies typically lead to poor boll retention, reduced fiber length, and diminished plant resilience to environmental stresses. Moreover, potassium has been shown to improve the plant’s capacity to withstand defoliation treatments, promoting better leaf abscission during harvest aid application. Interestingly, an excess of nitrogen can exacerbate potassium deficiencies, further impairing the plant’s response to harvest aids by disrupting the equilibrium of essential nutrients (Pettigrew et al., 2005).

The role of micronutrients, although required in trace amounts, is equally crucial for cotton’s physiological and reproductive success (Perumal et al., 2006). Zinc (Zn), manganese (Mn), and boron (B) are particularly influential in the plant’s response to harvest aids. Zinc is vital for enzyme activation and protein synthesis, with deficiencies often leading to poor boll development and delayed maturation. Manganese plays a significant role in photosynthesis, and a lack of manganese can result in chlorosis, affecting the plant’s overall vigor and ability to respond to defoliants. Boron is essential for cell wall formation and reproductive processes; boron deficiencies are often associated with reduced boll development, increasing the likelihood of boll drop and complicating the efficacy of harvest aids. The interactions between these micro- and macronutrients further emphasize the need for balanced nutrition to optimize cotton’s response to harvest aids (Guinn, 1982; Tariq et al., 2017).

Nutrient imbalances, particularly between nitrogen, phosphorus, and potassium, can also complicate the use of harvest aids. Excessive nitrogen can exacerbate deficiencies in potassium and magnesium, which are crucial for optimal boll development and fiber quality. This nutrient imbalance can result in poor boll maturation, decreased yield, and compromised fiber characteristics, further hindering the effectiveness of harvest aids. Moreover, the overapplication of phosphorus can sometimes result in potassium deficiencies, disrupting the plant’s ability to effectively utilize water and other nutrients. These interactions underscore the importance of precise nutrient management to ensure that cotton reaches the optimal physiological state for harvest aid applications, thus maximizing both the yield and fiber quality.

## 5 Conclusions and outlook

In conclusion, the use of harvest aids, including defoliants, in cotton cultivation provides several benefits: (1) it enhances the speed and efficiency of mechanical harvesting; (2) it facilitates centralized, one-off harvesting; (3) it enables early harvesting by mitigating adverse weather conditions, such as low temperatures; (4) it promotes leaf abscission and eliminates weeds, thus reducing fiber impurity rates during harvest; (5) when applied in fields with high planting densities and elevated humidity, it can prevent the rotting of lower cotton bolls due to pest and disease infestations; (6) it reduces the populations of pests such as thrips, bollworms, and yellow wilt disease during the harvest period, thereby decreasing fiber contamination and lowering the pest density in subsequent crops; and (7) it minimizes the impact of cotton’s indeterminate growth habits on the yield, accelerating the action of ethephon in promoting boll maturation and cracking, which increases the yield.

**Figure 4:**
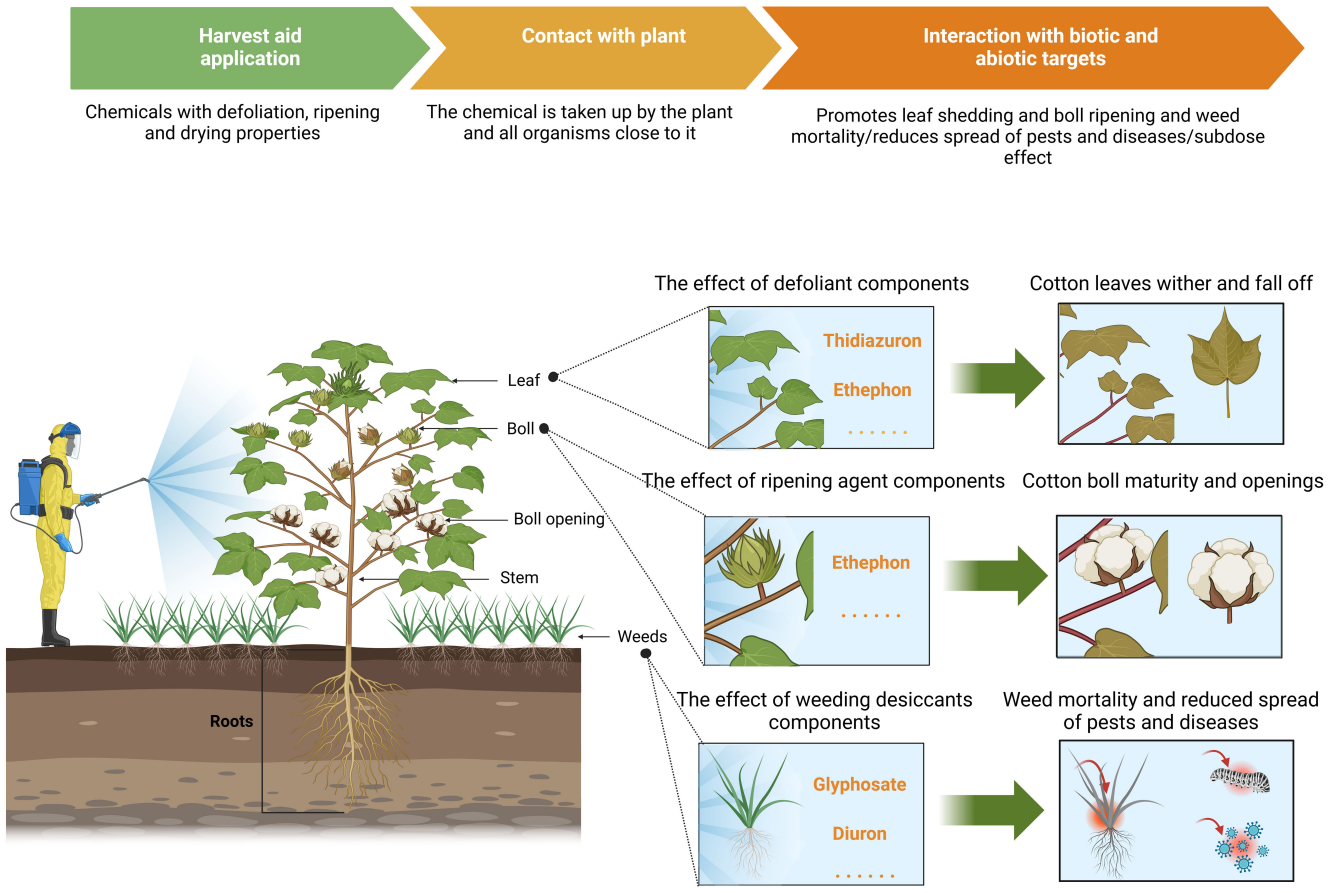
A schematic representation of the effect of chemical harvesting aids on defoliation and ripening in cotton.

It is important to highlight that cotton defoliation and ripening are exceedingly complex regulatory processes (see **Figure 4**) that are influenced by numerous factors, including regional climate conditions, agronomic management practices, and cultivated varieties. Adapting strategies to local conditions and applying targeted treatments constitutes a comprehensive “prescription.” Maintaining the normal developmental state of cotton plants and ensuring a balance between vegetative and reproductive growth are critical prerequisites. Once leaves abscise, the growth and development of the cotton bolls gradually diminish, until they ultimately cease. The objective of defoliation is to allow leaves to wither and detach from the plant. To accomplish this, defoliants should not instantly kill the leaves but allow them to remain viable long enough to form AZs. If leaves wither too rapidly, it may result in “withering without abscission,” thereby affecting the subsequent harvesting. Furthermore, the canopy structure at different spatial positions within cotton plants varies due to differing degrees of growth and development, leading to heterogeneous responses to harvest aids. Consequently, it is essential to uncover the patterns of these differential response mechanisms and develop compatible harvest aids and mechanical spraying equipment.

TDZ, the most commonly used chemical regulator for cotton defoliation and ripening, functions primarily through a mechanism involving the production of the endogenous hormone ethylene. However, the interactive mechanisms and regulatory signaling pathways of other plant hormones, such as ABA, gibberellins, auxins, and polyamines, in cotton defoliation and ripening remain poorly understood. For example, what are the similarities and differences between the natural senescence and maturation of cotton and TDZ-induced defoliation and ripening? Does the response to the AZ show synergistic interactions with these hormones? Furthermore, how do the upstream activators and downstream response elements of reactive oxygen species (ROS) signaling function and change during the TDZ-induced process? These questions require further investigation. Understanding the relationship between TDZ and plant hormones is essential for uncovering the underlying mechanisms of its action.

Currently, the agricultural sector is actively advancing into a new era of precision management technologies, integrating modeling, remote sensing monitoring, and robotic-assisted technologies into cotton cultivation. In this context, research that combines efficacy-oriented modeling with plant population characteristic-based remote sensing is increasingly significant. The former emphasizes the precise evaluation of the effectiveness of control measures, while the latter thoroughly investigates the overall impacts of regional environmental changes, cotton variety characteristics, cultivation management practices, and weed infestation patterns on the growth of cotton. Concurrently, modern gene editing technologies such as zinc-finger nucleases (ZFNs), transcription activator-like effector nucleases (TALENs), CRISPR/Cas9, base editing (BE), and prime editing (PE) are rapidly developing, providing robust technical support for precision agricultural management. In the foreseeable future, the following areas may emerge as key research directions:

(1). Intelligent Recognition and Localization Technologies: These technologies involve the use of advanced image recognition and machine learning technologies to develop systems or machine learning models that are capable of automatically identifying cotton leaves and bolls. Based on the cotton canopy maturity index, they can help determine the optimal defoliation time and location, thereby improving the defoliation efficiency and cotton harvest quality.
(2). Innovative Mechanical Harvesting Techniques: Traditional mechanical harvesting methods often result in some degree of fiber contamination. New mechanical harvesting technologies, such as using soft robotic arms or rotating brushes to simulate manual picking actions, aim to reduce the impurity rates of dead leaves and stem fuzz in cotton fibers while increasing the harvesting efficiency.
(3). Application of Biotechnology: This area involves exploring the use of biotechnologies, including gene editing and microbial engineering, to enhance the natural defoliation capabilities of cotton. By modifying or introducing specific genes, cotton can be engineered to shed its leaves more easily upon maturation, thereby reducing the reliance on chemical defoliants.

Lastly, future research should prioritize the long-term environmental impact assessment of cotton harvesting aids, particularly concerning the persistence and ecological implications of pesticide residues. Longitudinal environmental monitoring is essential to systematically evaluate the accumulation and potential bioaccumulation of these residues in soil and water and their subsequent entry into food chains. Additionally, it is critical to investigate whether the prolonged use of chemical harvest aids leads to significant residue accumulation in soils, as well as to elucidate whether herbicidal desiccants under hormesis effects exert lasting negative impacts on rhizosphere microorganisms and subsequent crops. Comprehensive assessments are required to clarify how these residues may ultimately affect other plants, animals, and human health through the food chain and food web interactions. Establishing comprehensive datasets from continuous field monitoring programs will be instrumental in understanding environmental impacts, facilitating accurate risk assessment, and guiding the sustainable use of cotton harvesting aids. Furthermore, interdisciplinary collaborations involving agronomy, environmental sciences, toxicology, and food safety are recommended to thoroughly elucidate potential ecological and human health risks and inform robust regulatory frameworks.

Regarding the use of harvesting aids in major cotton-producing countries (or regions), the adoption of chemical harvest aids (including defoliants, desiccants, and boll openers) closely correlates with the level of mechanization. In the United States and Australia, where mechanized harvesting is nearly universal, over 90% of cotton acreage typically employs one or more harvest aids—most commonly thidiazuron, diuron, and ethephon—to enhance the fiber quality and reduce green vegetation prior to machine picking. Brazil similarly shows high adoption rates (approximately 80–90%) in its primary mechanized production areas. In China’s Xinjiang region, where large-scale machine harvesting predominates, defoliants and boll openers are widely used, while in countries such as India and Pakistan—where manual harvesting still prevails—fewer than 15% of fields utilize harvest aids. However, despite these established patterns, comprehensive international data on harvest aid usage and active ingredient distribution remain limited. Most available information originates from national agricultural departments, industry associations, or smaller-scale academic studies, each with distinct methodologies and reporting standards, leading to potential inconsistencies and biases. Furthermore, precise data on per-hectare application rates, the relative proportions of various active ingredients, and regional adoption trends are often lacking or fragmented, leading to significant uncertainties. As a result, there is currently no fully integrated, globally harmonized database that captures both the extent of harvest aid use and the details of this across different agro-ecological zones. Researchers must therefore exercise caution when interpreting or comparing localized findings, as well as acknowledge the methodological constraints and variability that are embedded in existing datasets.
